# Mapping QTL for the traits associated with heat tolerance in wheat (*Triticum aestivum* L.)

**DOI:** 10.1186/s12863-014-0097-4

**Published:** 2014-11-11

**Authors:** Shyamal Krishna Talukder, Md Ali Babar, Kolluru Vijayalakshmi, Jesse Poland, Pagadala Venkata Vara Prasad, Robert Bowden, Allan Fritz

**Affiliations:** Forage Improvement Division, The Samuel Roberts Noble Foundation, Ardmore, OK 73401 USA; Department of Agronomy, University of Florida, Gainesville, Florida USA; Department of Agronomy, Kansas State University, Manhattan, KS 66506 USA; Department of Plant Pathology, Kansas State University, Manhattan, KS 66506 USA; USDA/ARS/Hard Winter Wheat Genetics Research Unit, Kansas State University, Manhattan, KS 66506 USA

**Keywords:** Wheat, Heat tolerance, GBS-SNP, Thylakoid membrane damage, Plasmamembrane damage

## Abstract

**Background:**

High temperature (heat) stress during grain filling is a major problem in most of the wheat growing areas. Developing heat tolerant cultivars has become a principal breeding goal in the Southern and Central Great Plain areas of the USA. Traits associated with high temperature tolerance can be used to develop heat tolerant cultivars in wheat. The present study was conducted to identify chromosomal regions associated with thylakoid membrane damage (TMD), plasmamembrane damage (PMD), and SPAD chlorophyll content (SCC), which are indicative of high temperature tolerance.

**Results:**

In this study we have reported one of the first linkage maps in wheat using genotype by sequencing SNP (GBS-SNP) markers to extreme response to post anthesis heat stress conditions. The linkage map was comprised of 972 molecular markers (538 Bin, 258 AFLPs, 175 SSRs, and an EST). The genotypes of the RIL population showed strong variation for TMD, SCC and PMD in both generations (F_10_ and F_9_). Composite interval mapping identified five QTL regions significantly associated with response to heat stress. Associations were identified for PMD on chromosomes 7A, 2B and 1D, SCC on 6A, 7A, 1B and 1D and TMD on 6A, 7A and 1D. The variability (R^2^) explained by these QTL ranged from 11.9 to 30.6% for TMD, 11.4 to 30.8% for SCC, and 10.5 to 33.5% for PMD. Molecular markers *Xbarc113* and AFLP AGCTCG-347 on chromosome 6A, *Xbarc121* and *Xbarc49* on 7A, *gwm18* and Bin1130 on 1B, Bin178 and Bin81 on 2B and Bin747 and Bin1546 on 1D were associated with these QTL.

**Conclusion:**

The identified QTL can be used for marker assisted selection in breeding wheat for improved heat tolerance in Ventnor or Karl 92 genetic background.

**Electronic supplementary material:**

The online version of this article (doi:10.1186/s12863-014-0097-4) contains supplementary material, which is available to authorized users.

## Background

Wheat is one of the most widely grown cereals globally. It possesses some adaptive plasticity which is the ability to exhibit some phenotypic change in responses to environmental conditions (i.e. high temperature). Even though there is adaptive plasticity, terminal heat stress has become a common limiting factor for almost all wheat grown in temperate regions, which accounts for 40% (36 million ha) of the total wheat production in the world [1,2]. The southern Great Plains of the USA is a temperate environment and accounts for 30-40% of US wheat production and often experiences temperatures of 32-35°C during grain filling stages [3]. Exposure to higher than optimum temperatures at this stage decreases yield and quality of wheat grain [4,5]. According to Wardlaw et al. [6], every 1°C rise above 15° to 20°C can cause a 3% to 4% yield reduction. Paulsen [7] reported that temperatures of 32° to 38°C can decrease wheat yield by 50% or more. The annual occurrence of moderate heat stress, accompanied by periodic extreme heat stress, prevents wheat from reaching its actual yield potential in these temperate regions [8].

Thermotolerance is a well-known adaptive phenomenon, which is induced by a short acclimation period at moderately high temperatures or by treatment with other non-lethal stress prior to subsequent heat stress. In the field, thermotolerance occurs under natural conditions and the effect of thermotolerance is an inherent component of heat tolerance [9]. Though high temperature is a frequently occurring phenomenon, relatively little is known about the critical genes controlling heat tolerance in plants [10]. To maintain growth and productivity, plants must adapt to stress conditions and exercise specific tolerance mechanisms. The alteration of various photosynthetic attributes under heat stress is a good indicator of heat tolerance as they show correlation with growth [11]. Injury to the photosystem can limit plant growth. Chlorophyll fluorescence, an indicator of photosystem II activity and thylakoid membrane damage, have been shown to correlate with heat tolerance [12]. It has been reported that wheat genotypes with higher variable fluorescence (F_v_) have higher yield potential. Maximum (F_m_), base (F_0_), variable fluorescence (F_v_), and half-time between F_0_ and F_m_ have been reported to have strong genetic correlation with grain yield of durum wheat [13]. Plasma membrane stability (also called cell membrane thermostability), which is the reciprocal of plasma membrane damage, is related to cellular thermotolerance. Increased permeability of membranes is evidenced by increased loss of electrolytes, an indication of decreased membrane stability and has been used as an indirect measure of heat-stress tolerance in diverse plant species, including wheat [14], sorghum (*Sorghum bicolor* L. Moench) [15], and barley (*Hordeum vulgare* L.) [16]. Membrane thermostability has been reported to have a strong genetic correlation with grain yield in wheat [1,9]. Marsh et al., [17] found a large portion of the variability for membrane stability to be controlled by a small number of genes. Heritability of membrane thermostability in maize (*Zea mays* L.) was estimated to be 73% [18].

In spite of being promoted as a promising breeding tool, the use of membrane thermostability and chlorophyll fluorescence for improvement of thermotolerance in wheat is very limited because of time-consuming and labor intensive field evaluation processes. Membrane stability requires destructive sampling and there is large potential for error inherent in the process of estimating membrane stability. Similarly, measurements of chlorophyll fluorescence require use of expensive instrumentation and, in some cases, necessitates dark adaptation of the leaf tissue, which limits the number of plants that can be screened in a given day. In addition to the complex estimation processes, these traits are influenced by environmental conditions. Thus, improving heat tolerance through traditional breeding methods is difficult. Identification of DNA markers associated with acquired thermotolerance would allow marker assisted selection and increase the efficiency for improving these traits through breeding. In addition, the identification of QTL would be useful in the identification of genes that are important for tolerance to heat stress.

Heat tolerance is a quantitative trait [12,19]. Despite its importance, only a few QTL mapping studies have focused on heat tolerance. Yang et al. [19] found QTL linked to grain filling duration on the short arms of chromosomes 1B and 5A. In addition, QTL for heat tolerance under hot and dry conditions were detected on chromosomes 2B and 5B in a spring wheat population [20]. In another study, conducted under short-term reproductive stage heat stress, several QTL were found on chromosome 1A, 1B, 2A, 2B, 3B, 5A and 6D for heat susceptibility indices of various morphological and yield traits [8,21]. Paliwal et al. [22] reported QTL for thousand grain weight, grain fill duration and canopy temperature depression on chromosome 2B, 7B and 7D, respectively. Vijayalakshmi et al. [23] reported QTL with significant effects on grain yield, grain weight, grain filling, stay green and senescence associated traits on 2A, 3A, 4A, 6A, 6B and 7A under post-anthesis high temperature stress in wheat. Most of the reported QTL maps have been based on low density SSR and/or AFLP markers. Developing a map with high density molecular markers is needed in order to get a better understanding of the architecture of complex traits. Genotype-by-Sequencing (GBS) is an approach to develop SNP markers which can be used for mapping traits in diverse organisms. This approach is very simple and cost effective and is based on high throughput next generation sequencing. In this method, SNPs are discovered by sequencing a subset of genomic fragments following the use of restriction enzymes [24,25].

In this study we used the same population and marker data of Vijayalakshmi et al. [23] along with an additional set of Bin markers (SNPs data from one bin considered as a haplotype and referred to as single SNP) developed by using the Genotype By Sequencing (GBS) approach. The objectives of the present study were to increase the marker density in the population and identify QTLs associated with different traits, thylakoid and plasma membrane and chlorophyll damages, which provide heat tolerance in wheat.

## Methods

### Genetic materials and growth conditions

Ventnor, a hard white Australian wheat, and Karl 92, a hard red winter wheat from Kansas were crossed to develop a recombinant inbred line population (RIL). The pedigree of Ventnor is unknown, while Karl 92, is an F_11_ reselection from the cultivar Karl. The pedigree of Karl is PlainsmanV/3/Kaw/Atlas 50//Parker*5/Agent [26]. Ventnor has been shown to have superior heat tolerance based on its ability to maintain photosynthetic capacity and kernel weight when exposed to post anthesis heat stress [19,27,28], while Karl 92 was found moderately sensitive to post anthesis heat stress. The recombinant inbred line population was developed by advancing from the F_2_ through single seed descent (SSD) in the greenhouse to generate a set of F_6:7_ RILs [23]. The entire population was characterized for thylakoid and plasma membrane damage, and for chlorophyll content in the F_6:9_ and F_6:10_ generations under optimum (20/15 ± 2°C day/night temperature) and high temperature stress (36/30 ± 1°C) conditions during the post anthesis stage.

The plants were grown in a greenhouse at an optimal temperature of 20/15 ± 2°C day/night temperature with 16 h photoperiod and light intensity of 420 μmol m^−2^ s^−1^ approximately. Fertilizer, systematic insecticide, and fungicide were applied as needed to avoid any malnutrition or biotic stresses. Plants were watered as needed to avoid any stress. Each line was planted in six pots with three plants per pot. The primary tiller of each plant was tagged at anthesis and was used for estimating physiological traits. Eight days after anthesis (at full anthesis), plants were transferred to a controlled growth chamber maintained at optimum growth conditions. Due to the genetic variation for anthesis, genotypes were grouped based on their similar anthesis date (±1 day) and exposed to the high temperature. Once genotypes were moved from the greenhouse to growth chamber, six pots/line were divided into two growth chambers (3 pots/chamber) and chambers were maintained optimum temperature condition (20/15 ± 1°C) for 48 h to facilitate adaptation to growth chamber conditions. At ten days after anthesis, one growth chamber was left under optimum temperature conditions, while the plants in the other one were subjected under high temperature stress.

### Heat treatment and physiological characterization

The controlled chamber was maintained at 20/15 ± 1°C with 16-h photoperiod, and 420 μmol m^−2^ s^−1^ light intensity. On the other hand, the temperature in the high temperature growth chamber was raised from 20/15 ± 1°C to 36/30 ± 1°C with adequate moisture over a 48 h period and remained for duration of 10 d. The intensity of light was 420 μmol m^−2^ s^−1^. Water was provided to plants as needed in both the control and heat treated conditions. Each of the three pots of a genotype was treated as biological replications. Pots were randomly arranged inside the growth chamber for both the controlled and heat treated conditions.

Chlorophyll a fluorescence, the ratio of variable (F_v_) to maximum fluorescence (F_m_), was used as an indirect method to assess thylakoid membrane damage [29,30]. F_v_/F_m_ was measured on intact flag leaves one third of the way from the base of the abaxial surface after 1 h of dark adaptation. Fluorescence was measured using a pulse modular fluorometer (Model OS5- FL, Opti-Sciences, Hudson, NH, USA) in both the control and heat treated plants at 4-, 7-, and 10-d after heat treatment. In each treatment (control and heat treated growth chamber), F_v_/F_m_ was measured from three flag leaves (3 different plants) for each biological replication. An average of three measurements was used to estimate thylakoid membrane damage (TMD). Thylakoid membrane damage (TMD) due to heat stress was assessed by comparing F_v_/F_m_ values between control and heat treated plants. The relative damage was estimated as follows: % TMD = [((F_v_/F_m_-heat)-(F_v_/F_m_-control))/(F_v_/F_m_-control)]*100. As the %TMD values were calculated in percentage, to increase homogeneity of the data, the percent values were transformed by Log_2_ function. The Log_2_ transformed data were used for statistical analysis.

A self-calibrating SPAD chlorophyll meter (Model 502, Spectrum Technologies, Plainfield, IL) was used to measure chlorophyll content. Chlorophyll content was measured from the same flag leaves and leaf blade areas where fluorescence measurements were taken at 4-, 7-, and 10-d after heat treatment. In each treatment (control and heat treated growth chamber), chlorophyll contents were measured from three flag leaves (3 different plants) for each biological replication. An average of three measurements was used to represent chlorophyll content for statistical analysis.

Plasma membrane damage (PMD) was assessed using the method described by Ristic and Cass [31]. Leaf disks (diameter = 5 mm) were collected from two individual flag leaves at two different plants within each biological replication at 7- and 10-d after heat treatment and placed in de-ionized water (4 ml) in sealed vials. The vials were stored overnight on a shaker at 5°C. Electroconductivity of the aqueous solution was measured with a Metter Toledo (SevenMulti S70) conductivity meter. The tissue samples were then autoclaved. The conductivity of the solution was again measured after storing the samples on a shaker at 5°C overnight. The percent electrolyte leakage was calculated based on the conductivity before and after autoclaving. The average value of two flag leaves within each biological replication was used to estimate % PMD. The percent damage was calculated as 100 × (% leached_h_ -% leached_c_)/(X-% leached_c_), where h was stressed, c was control, and ‘X’ was % leached value corresponding to 100% damage which was assumed to be 100% leached. As the PMD values were calculated in percentage, to increase homogeneity of the data, the percent values were transformed by Log_2_ function. The Log_2_ transformed data were used for statistical analysis.

Adjusted mean (Best Linear Unbiased Prediction, BLUP) values were estimated for each sampling date of chlorophyll content, log transformed TMD and PMD data across two generations. The estimated adjusted mean values of each sampling date were used for QTL analysis.

### Statistical analyses

The mean values over three time points (4-, 7-, 10-d) for TMD and SCC, and two time points (7- and 10-d) for PMD were used for analysis of variance (ANOVA) to determine the main effects of genotype (RIL), block, and replication factors. During analysis, growth chambers were used as blocks. Analyses of variance and least square means of all traits were estimated using the SAS PROC MIXED procedure. Phenotypic correlations and simple regression were calculated for all traits using Microsoft Excel. Adjusted mean (Best Linear Unbiased Prediction, BLUP) values were estimated using the R v2.12.0 statistical programming language [32].

### Molecular markers and map development

A total of 972 molecular markers were used in the mapping effort and included 538 Bin, 258 AFLPs, 175 SSRs, and an EST. The detailed description of the AFLP, SSR and EST markers has been provided by Vijayalakshmi [33] and Vijayalakshmi et al. [23]. Bin markers were developed using a genotype by sequencing (GBS) approach [25]. DNA was isolated from F_10_ plants leaves and digested by HF-*PstI* (High- Fidelity) and *MspI* (New England BioLabs Inc., Ipswich, MA 01938) followed by ligation with a set of 96 adapters (adapter 1) combined with a common adapter (Y adapter) in every reaction. Ligated samples were pooled in a single tube followed by PCR amplification to produce a single library from 96 samples. That library was sequenced on a single lane of an Illumina HISEQ 2000. Barcodes allowed assignment of Illumina raw data to individual samples. Sequences were trimmed to a 64 bp read and SNP-calling was performed using a custom script in Java (www.maizegenetics.net, sourceforge.net/projects/tassel/). To reduce the ratio of missing data, all co-segregating SNPs in a bin were called as bin marker. The reference sequences of SNPs, bin compositions, and marker segregation data have been provided in Additional file 1. JoinMap ver. 4.0 [34] with the Kosambi function [35] was used to assemble AFLP, SSR, EST and bin markers into a linkage map at LOD score 5.0. Significantly distorted markers were excluded from the analysis during the group preparation. All linkage maps are provided in Additional file 2.

### Quantitative trait locus (QTL) analyses

The Windows version of QTL Cartographer V2.5 [36] was used to conduct composite interval mapping (CIM) analysis based on model 6. The forward and backward regression method was used as a cofactor to control the genetic background while testing a position in the genome. The walking speed chosen for the QTL analysis was 2.0 cM. QTL were verified by LOD scores (2.88-3.28) compared to the threshold calculated from 1000 permutations for p < 0.05. We also accepted those QTL as significant at a LOD value of 2.5 or more, once it fulfilled the declaration criteria and co-localized with other traits described by Paliwal et al. [22] and Pinto et al. [37]. QTL names were designated following the International Rules of Genetic Nomenclature (http://wheat.pw.usda.gov/ggpages/wgc/98/Intro.htm).

### Epistasis analysis

QTLs with epistatic effect were detected by QTL IciMapping V4.0 [38] selecting ICIM-EPI with a probability value for entering variables (PIN) of 0.0001. The default threshold LOD of 3.0 for ICIM-EPI was used to detect epistatic QTLs.

## Results

### Genetic variations, physiological changes and assessment of heat tolerance

The variance components associated with different effects are presented in Table 1. The genotypes showed strong variation for TMD, SCC and PMD in both generations (F_10_ and F_9_) of the RIL population. The variance component associated with genotypes contributed more than 92% (deduced from Table 1) of total variation.

**Table 1:** **The variances of thylakoid membrane damage (TMD), SPAD chlorophyll content (SCC), and plasma membrane damage (PMD) under post-anthesis high temperature stress over two generations of RILs**

**Sources of variation**	**DF**	**F**_**10**_ **generation**			**F**_**9**_ **generation**		
		TMD	SCC	PMD	TMD	SCC	PMD
Block	10	0.004	1.600	0.040	0.015	2.080	0.100
Rep (block)	22	0.000	0.000	0.0004	0.0003	0.000	0.000
Genotypes	102	0.900	52.7	1.22	0.901	53.20	1.04
Residual	174	0.060	0.720	0.010	0.060	0.760	0.070

DF = degrees of freedom.

**Figure 1:**
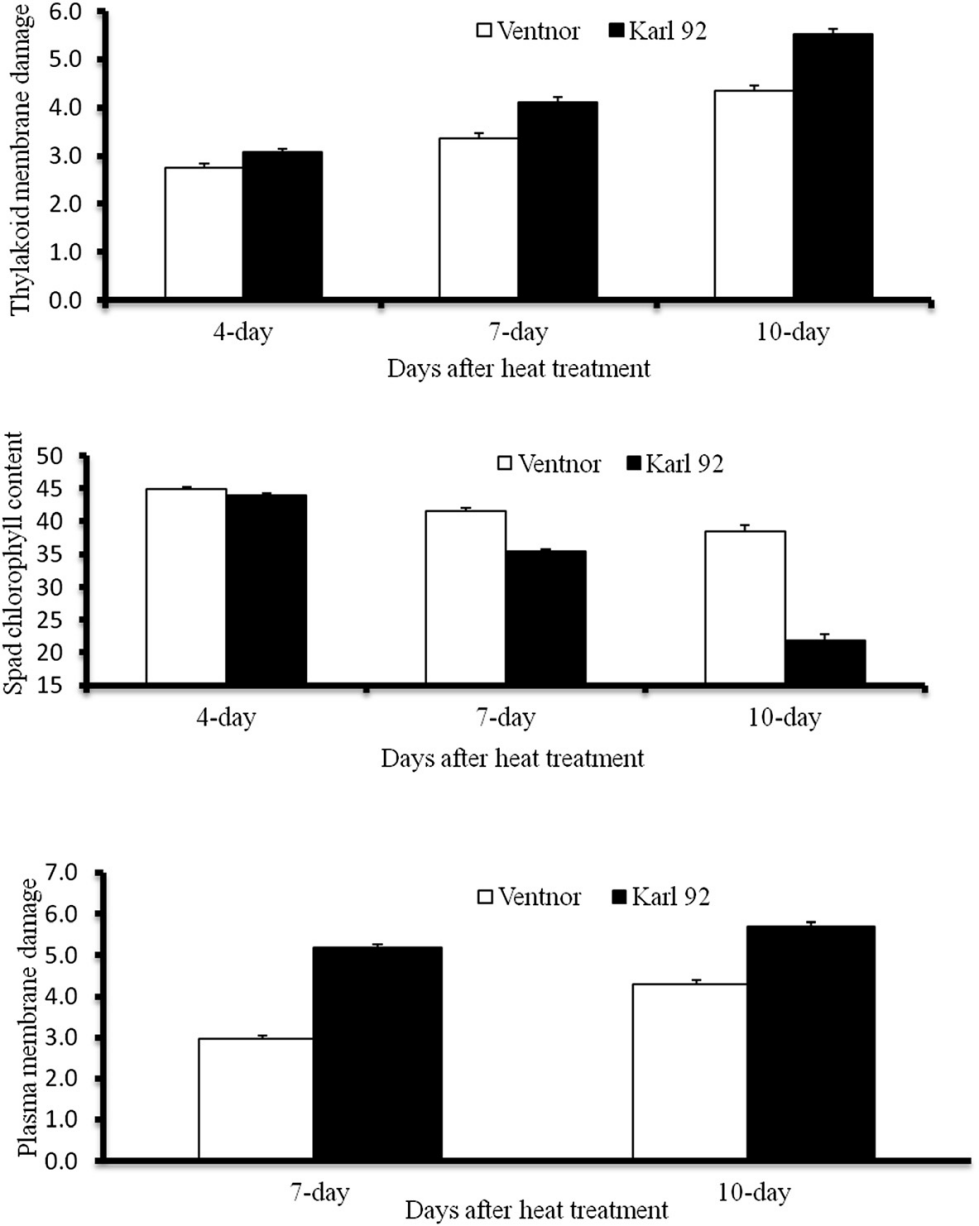
**Mean comparison of tolerant (Ventnor) and sensitive (Karl 92) parents at different times after heat treatment.** TMD represents thylakoid membrane damage; SSC represents SPAD chlorophyll content; PMD represents plasma membrane damage.

**Figure 2:**
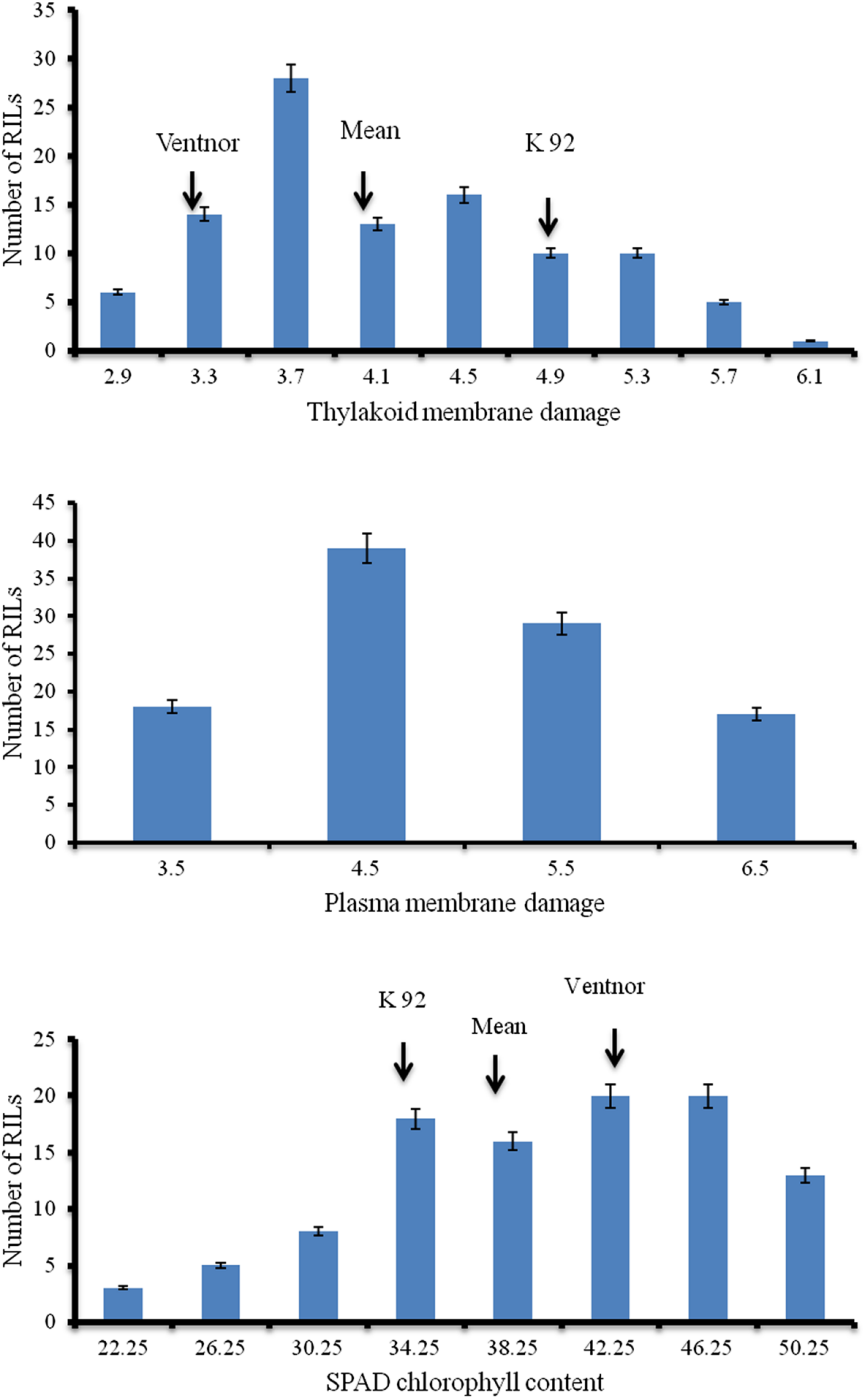
**Frequency distributions of mean thylakoid membrane damage (TMD), SPAD chlorophyll content (SCC) and plasma membrane damage (PMD) for 101 RILs.**

The mean values of TMD, SCC and PMD for parents and progeny under heat stress are shown in Figure 1 and 2. The data indicate that the increased exposure to heat stress increases damage to the plasma membrane, thylakoid membrane, and reduces chlorophyll content in the heat stressed plants in both the tolerant and sensitive parents, however, the damage was lower in the tolerant parent than in the sensitive parent (Figure 1). Compared to the control, mean TMD values ranged from 12.2% in Ventnor to 32.1% in Karl 92, and mean PMD ranged from 14.3% in the tolerant parent to 42.8% in the sensitive parent (data not presented). The average value of SCC under heat stress (not compared with control) ranged from 43.3 in the tolerant parent to 30.6 in the sensitive parent (data not presented). The mean values for TMD, SCC and PMD in the F_9_ and F_10_ generations were 21.9% and 24.8%, 38.9% and 36.5%, and 28.6% and 32.9%, respectively (calculated from non-transformed data). The distribution of values for TMD, PMD and SCC are presented in Figure 2. Both positive and negative transgressive segregation were observed for both TMD and SCC (Figure 2), as well as for PMD (transgressive segregation not shown).

**Figure 3:**
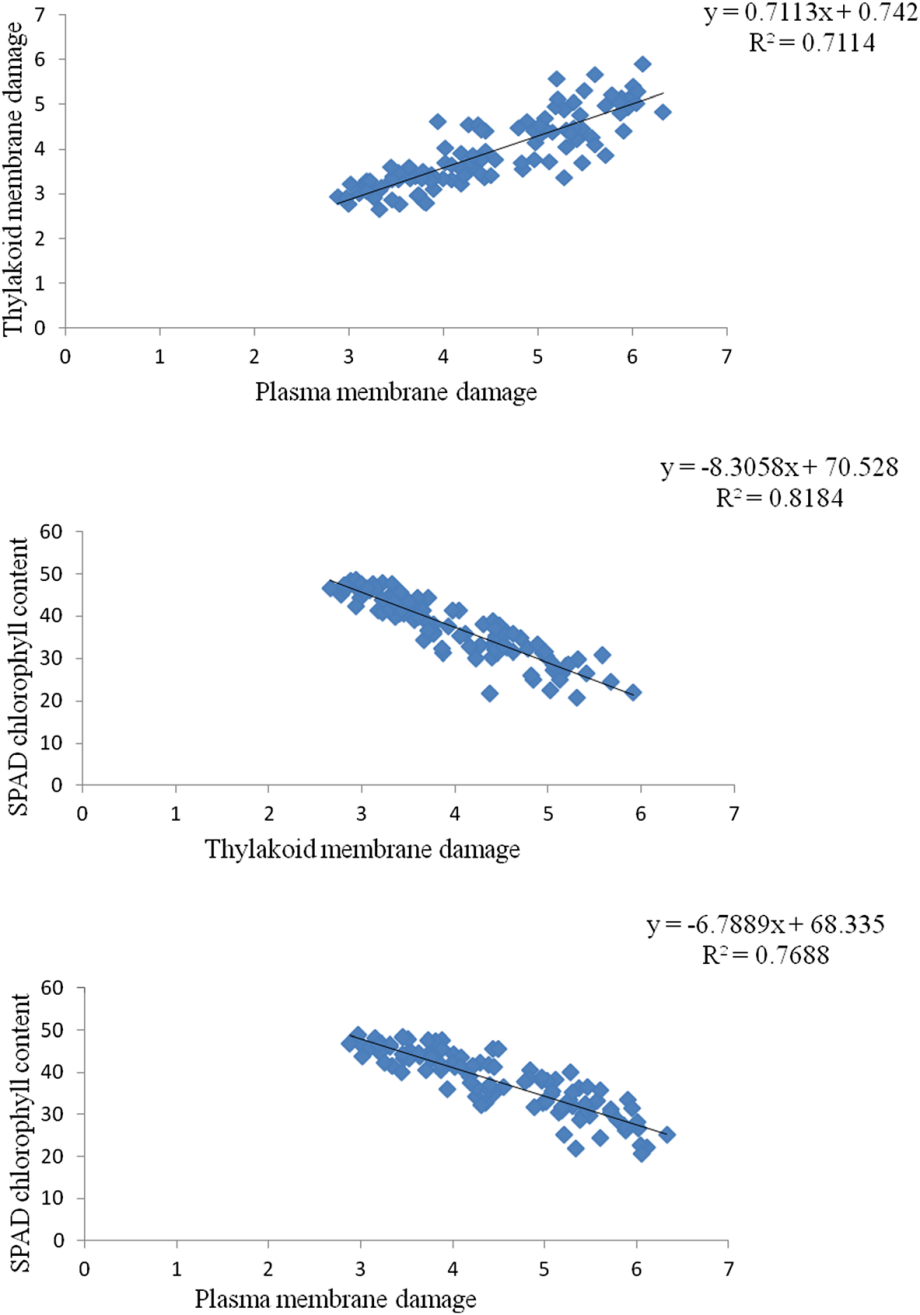
**Relationships among thylakoid membrane damage (TMD), SPAD chlorophyll content (SCC) and plasma membrane damage (PMD) in the RIL population.**

Very strong phenotypic associations were observed among the three traits (Figure 3). SCC explained 82% and 76% variability in TMD and PMD, while TMD explained 71% of the variability in PMD. Although all traits were very strongly associated, the association between TMD and SCC was higher than the association between those two traits and PMD. These strong relationships suggest that the three traits might be under similar genetic control and are physiologically related.

### Molecular markers and linkage map

Five hundred sixty of 972 markers were used to produce sizeable linkage groups. Linkage groups without any SSR markers were not considered a viable group in this analysis. Of the 560 group-forming markers, SSRs accounted for 91, Bin markers accounted for 391 and AFLPs accounted for 78. The rest of the markers were ungrouped, grouped without an SSR (it cannot be assigned to a chromosome), or distorted. Twenty-two linkage groups were identified and covered a total length of 1044 cM, with an average interval of 1.86 cM between markers. All chromosomes except 5D were represented in the linkage groups. Chromosome 2A and 7D each had two groups. Comparing across genomes, the maximum number of markers mapped to the B genome (45.44%), followed by the A genome (42.68%), and the D genome (11.96%). Density of markers was greatest on chromosome 1B, with an average distance of 0.81 cM between markers, and least on chromosome 2D, with an average interval of 3.25 cM between markers.

### QTL analysis

**Figure 4:**
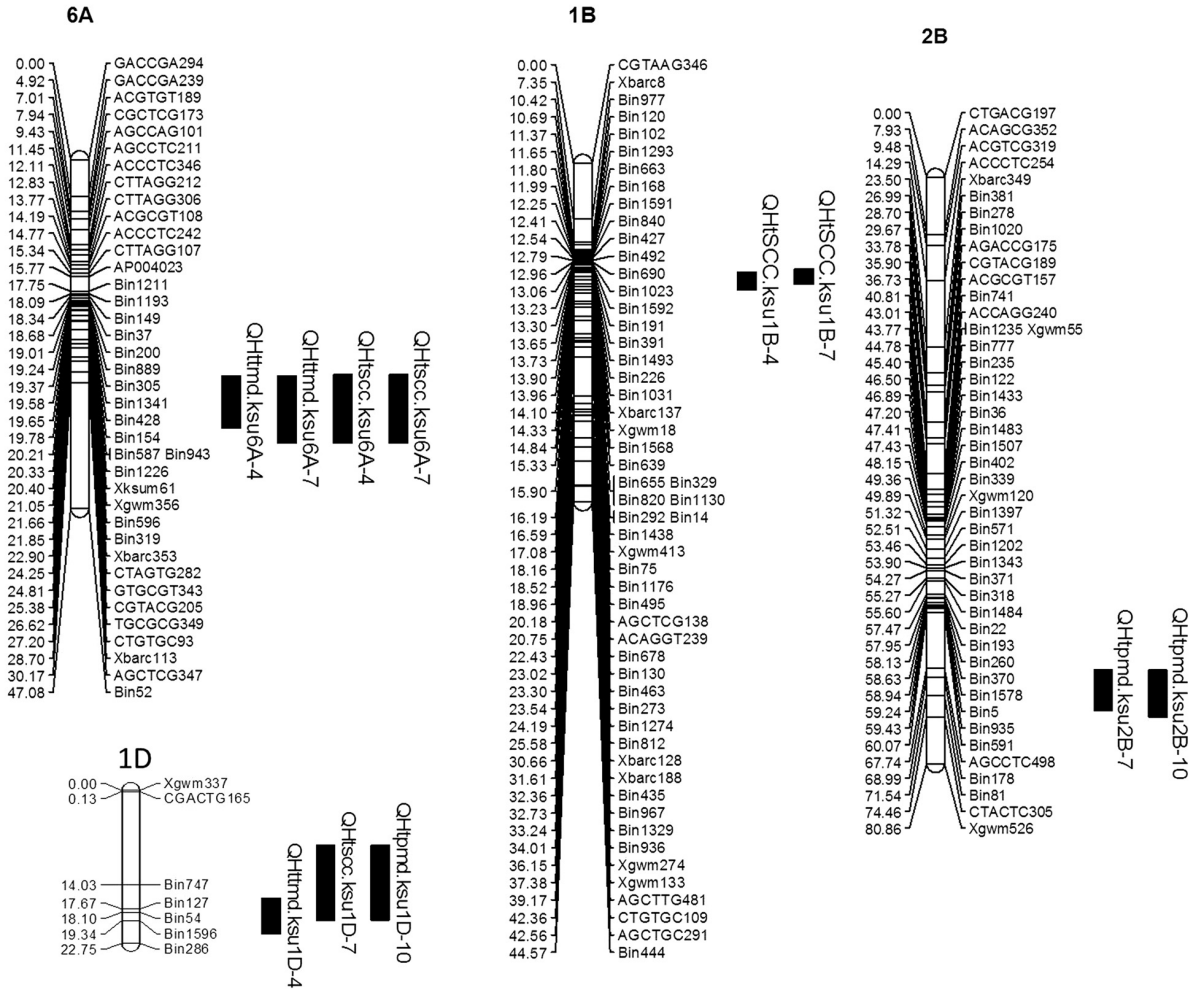
**Primary genomic regions of heat stress tolerance QTL on 6A, 1B, 2B and 1D identified by composite interval mapping in a Karl 92 x Ventnor RIL population.** TMD represents thylakoid membrane damage; SSC represents SPAD chlorophyll content; PMD represents plasma membrane damage.

**Figure 5:**
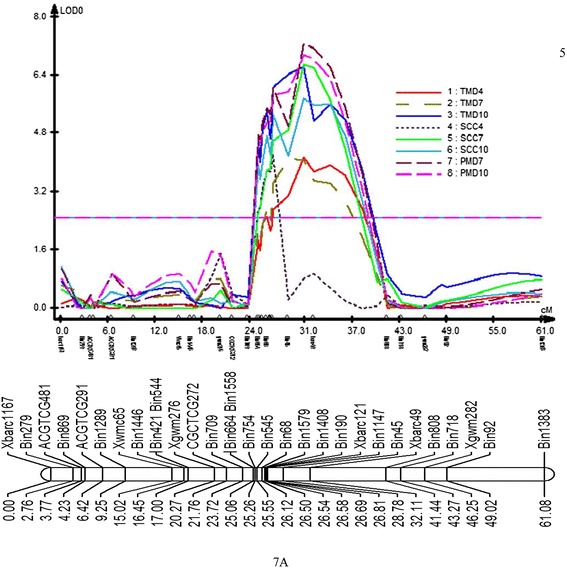
**Likelihood plots obtained by composite interval mapping for QTL mapped on chromosome 7A.** TMD represents thylakoid membrane damage; SSC represents SPAD chlorophyll content; PMD represents plasma membrane damage. The horizontal line represents a LOD value of 2.5.

Five genomic regions (chromosome 6A, 7A, 1B, 2B and 1D) were associated with a significant number of QTL. The QTL were associated with LOD scores ranging from 2.5 to 7.28 and explained from 10.53% to 33.51% of the phenotypic variability (Table 2, Figure 4 and 5). The QTL on chromosome 6A was associated with SCC and TMD in the first two sampling dates. In all cases, the QTL was flanked by markers *Xbarc113* and AGCTCG347. Presuming this represents one QTL affecting multiple traits, on an average it explained 16.48% of the phenotypic variation for SCC and 13.39% of TMD in the first two sampling dates (4- and 7- d after heat treatment) (Table 2 and Figure 4).

**Table 2:** **Chromosomal locations, QTL length, determination coefficients (R** ^**2**^ **), additive effects and LOD values for significant QTL in Karl 92/Ventnor 101 recombinant inbred line (RIL) population**

**Trait**	**Chrom**	**QTL**	**Flanking marker**	**Length (cM)**	**LOD**	**AD**	**R** ^**2**^
TMD4	6A	*QHttmd.ksu-6A*	*Xbarc113, AGCTCG347*	6.98	2.58	−0.19	11.90
TMD7	6A	*QHttmd.ksu-6A*	*Xbarc113, AGCTCG347*	8.98	3.21	−0.29	14.87
SCC4	6A	*QHtscc.ksu-6A*	*Xbarc113, AGCTCG347*	9.18	3.8	1.42	17.57
SCC7	6A	*QHtscc.ksu-6A*	*Xbarc113, AGCTCG347*	9.18	3.32	2.34	15.38
TMD4	7A	*QHttmd.ksu-7A*	*Xbarc121, barc49*	11.12	4.15	−0.24	19.15
TMD7	7A	*QHttmd.ksu-7A*	*Xbarc121, barc49*	9.32	4.08	−0.24	18.86
TMD10	7A	*QHttmd.ksu-7A*	*Xbarc121, barc49*	13.05	6.66	−0.48	30.62
SCC4	7A	*QHtscc.ksu-7A*	*Bin754, Bin45*	3.72	4.22	1.53	19.53
SCC7	7A	*QHtscc.ksu-7A*	*Xbarc121, barc49*	11.42	6.7	3.53	30.84
SCC10	7A	*QHtscc.ksu-7A*	*Xbarc121, barc49*	11.42	5.73	4.9	26.59
PMD7	7A	*QHtpmd.ksu-7A*	*Xbarc121, barc49*	13.05	7.28	−0.50	33.51
PMD10	7A	*QHtpmd.ksu-7A*	*Xbarc121, barc49*	13.05	6.95	−0.46	32.03
SCC4	1B	*QHtscc.ksu-1B*	*gwm18, Bin1130*	2.30	2.5	1.07	11.37
SCC7	1B	*QHtscc.ksu-1B*	*gwm18, Bin1130*	2.0	2.75	2.01	12.63
TMD4	1D	*QHttmd.ksu-1D*	*Bin747, Bin1596*	5.31	3.06	−0.18	14.12
SCC7	1D	*QHtscc.ksu-1D*	*Bin747, Bin1596*	11.21	3.58	2.5	16.64
PMD10	1D	*QHtpmd.ksu-1D*	*Bin747, Bin1596*	11.21	2.52	−0.28	11.59
PMD7	2B	*QHtpmd.ksu-2B*	*Bin178, Bin81*	5.55	3.22	−0.30	10.53
PMD10	2B	*QHtpmd.ksu-2B*	*Bin178, Bin81*	6.47	3.75	−0.31	17.22

TMD, thylakoid membrane damage; SCC, SPAD chlorophyll content; PMD plasma membrane damage. AD, additive effect. For TMD and PMD, negative value of AD, and for SCC, positive value of AD indicates the Ventnor allele having a positive effect on the trait.

The QTL on the long arm of 7A showed significant effects for all three traits across all three sampling dates. This QTL was flanked by the *Xbarc121* and *Xbarc49* markers. It explained 19.15% to 30.62% of the variability for TMD, 19.53% to 30.84% of the variability for SCC, and 32.03 to 33.51% of the variability for PMD (Table 2 and Figure 5). This QTL was the most consistent across all traits and explained the highest proportion of phenotypic variability. It showed significant effects in all sampling dates for TMD and SCC (4-, 7- and 10-d after heat treatment), and for PMD (7- and 10-d after heat treatment).

The QTL on chromosome 1B was associated with the first two sampling dates (4- and 7- d after heat treatment) of SCC and flanked by *gwm18* and Bin1130 (Table 2 and Figure 4). There was a 0.39 cM displacement between the regions identified for two sampling days.

The QTL identified on chromosome 2B was flanked by Bin 178 and Bin 81 and was significant for both sampling dates for PMD (7- and 10-d after heat treatment). This QTL explained an average of 13.88% of the phenotypic variation for PMD and was remarkably consistent in its effect (Table 2 and Figure 4).

The fifth QTL identified was on chromosome 1D. This QTL was significant for PMD in the latest sampling date (10 d after heat treatment), SCC in the middle date (7 d after heat treatment), and TMD in the earliest date of heat treatment (4 d after heat treatment). The highest phenotypic variability was for SCC (16.64%) followed by TMD (14.12%) and then PMD (11.59%). The QTL was flanked by markers Bin 747 and Bin 1596. Heat tolerant alleles for PMD, SCC and TMD of all the reported QTL were contributed from the tolerant parent Ventnor (Table 2).

**Figure 6:**
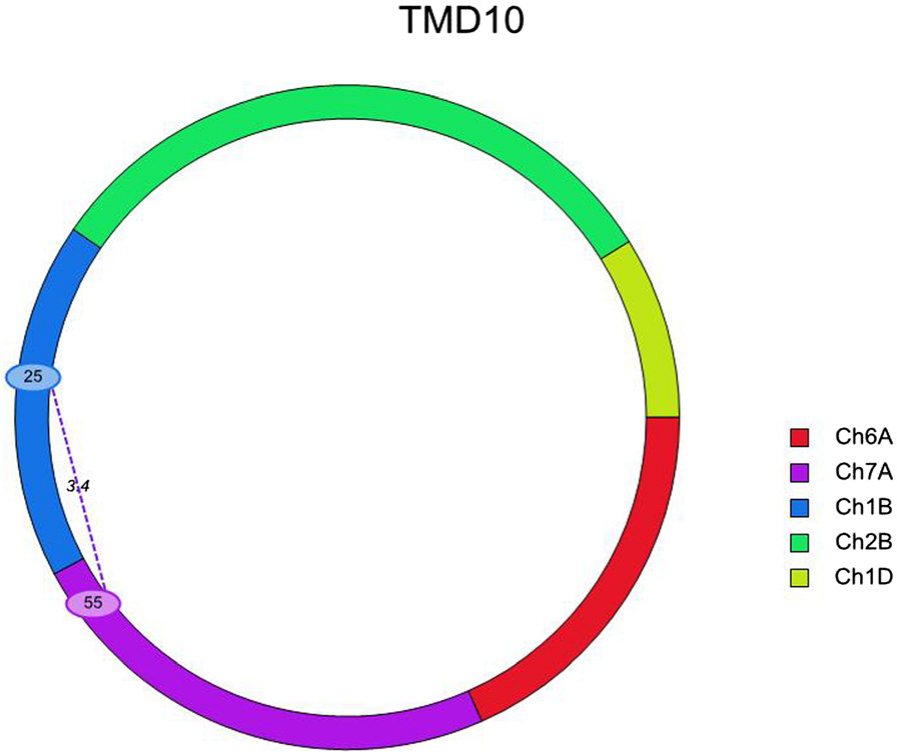
**Epistasis QTL between chromosome 7A and 1B for TMD at 10 d.** TMD represents thylakoid membrane damage; Ch1A, Ch1B, Ch2B, Ch6A and Ch7A represents. Chromosome 1A, 1B, 2B, 6A and 7A respectively; The number on the chromosomal region denote the QTL position. The number on the linking line (3.4) represents the LOD value of the QTL.

A significant epistatic QTL was detected between chromosome 7A and 1B for TMD at 10 d (Figure 6). Other traits with various time points did not show any epistasis.

## Discussion

In this research SPAD chlorophyll content and chlorophyll fluorescence (F_v_/F_m_) measurements were used to estimate chlorophyll content and thylakoid membrane damage of heat stressed plants. Loss of chlorophyll content during grain filling was already reported to be associated with reduced yield under the field conditions [1]. Various authors have also found thylakoid and plasma membrane damages were associated with grain yield [1,12-16,39]. In our study, we found strong correlation among these traits which indicate that these traits might be under pleiotropic genetic control. Despite strong correlations, we found some variability in QTL regions (linkage group 1B and 2B) for these traits along with some common QTLs (linkage group 6A, 7A and 1D). This might be because of polygenic inheritance of those traits, difficulty of estimation of these traits and 25-30% variabilities among these traits which are not common (deduced from Figure 3). As a result, measuring more than one trait will provide more precise information. However, considering limited resources in the plant breeding programs, SCC could be used to produce reasonable information for heat tolerance as it showed stronger correlations with PMD and TMD than the correlation between PMD and TMD itself.

This study was conducted using the same population and marker data of Vijayalakshmi et al. [23], along with an extra set of GBS SNP markers. Present study has developed one of the earliest wheat linkage maps by using GBS SNP marker for QTL study under heat stress conditions. The additional markers, and possibly, the use of different mapping software resulted in some variability in linkage group formation compared to Vijayalakshmi et al. [23]. The 2A and 6A groups of Vijayalakshmi et al. [23] were fused together and were assigned to 6A in this study. They assigned the 2A group based on *Xgwm356* and *Xbarc353*. Those markers have been reported to map to both 2A and 6A (http://wheat.pw.usda.gov/GG2/index.shtml). In our study, the additional markers allowed the identification of a single linkage group. Two markers specific to 6A allowed a more accurate chromosomal assignment.

Vijayalakshmi et al. [23] and the current study used the same mapping population, but varied in the method of exposing the plants to the stress and to the traits evaluated. We exposed plants to moderate temperature to develop thermotolerance before the chronic heat treatments. However, some QTL regions were common in both studies, though the studies were conducted under different temperature regimes. The similarity of findings increases confidence that these chromosomal regions are truly associated with heat tolerance in this population. These genomic regions might have certain genes which could be manipulated by wheat breeders to improve heat tolerance In our study, five genomic regions (6A, 7A, 1B, 2B and 1D) were significantly associated with traits related to heat tolerance. Of nineteen QTL identified in this study, twelve QTL explained greater than 15% of the phenotypic variability and should be considered as major QTL. Whereas, Vijayalakshmi et al., [23] found several QTL on chromosomes (2A, 3A, 5A, 6A, 7A, 3B, 4B, 6B, 7B and 5D ) for different senescence related traits under high temperature stress (32/25°C day and night temperature from 10 d after anthesis to physiological maturity). QTL for 75% stay-green on chromosomes 2A and 3B and a region on 2A for 25% stay-green were not found in the current work.

We observed that the QTL on 1D was associated with all three traits making it a potentially important QTL for heat tolerance. Pinto et al. [37] reported QTL for days to anthesis on 1D under drought and temperate irrigated conditions. This QTL was likely not identified in the Vijayalakshmi et al. [23] study because the markers associated with the trait are new SNPs. The addition of new SNPs might also be the reason we were able to identify QTL on 1B in this study. Pinto et al. [37] in their study reported several QTL on 1B including QTL for canopy temperature, yield, and chlorophyll content in the grain filling stage. The 2B QTL was also not identified in the Vijayalakshmi et al. [23] study due to the lack of markers in the region. The only trait associated with 2B in the present study is PMD. It may be that this locus is only associated with membrane stability and not with photosynthetic function. Mason et al. [21] reported a stable QTL on 2B for heat susceptibility index (HIS) of grain number.

The QTL identified on chromosome 6A was significant, consistent and co-localized for SCC and TMD across the first two sampling dates (4- and 7- d after heat treatment). Vijayalakshmi et al. [23] found two QTL on 2A between *Xgwm356* and CGT.TGCG-349, and between CGT.TGCG-349 and CTCG.ACC-242 for 75% stay-green, 25% stay-green, 50% stay-green, maximum rate of senescence, and time for maximum senescence. Other markers associated with QTL on 2A and 6A in their study were *Xgwm353*, GTGACGT-189, GTGCTA-282 and CGACGCT-173. In our study, chromosome 6A encompasses both 2A and 6A of Vijayalakshmi et al. [23]. Most of the trait-associated markers from that study are present in the interval region of our putative QTL. As a result, we believe that the QTL on 6A is the same QTL previously identified on 2A and 6A and is associated with stay-green related traits under high temperature.

The QTL on chromosome 7A was very consistent for all three traits across all the sampling dates with very high LOD values (Table 2). Phenotypic variability explained by this QTL was also very high and ranged from 18.86% to 33.51%. It was flanked by marker *Xbarc121* and *Xbarc49*. Vijayalakshmi et al. [23] reported a QTL on 7A for F_v_/F_m_ and time to maximum rate of senescence (TMRS) associated with marker *Xbarc121. Xbarc49* marker was physically mapped to 7A by Sourdille et al. [40] on wheat deletion bin C7AL 1–0.39. EST WHE2105_F08_K15ZS was also located in that bin and was found to be similar to the stress responsive gene (*srg6*) in *Hordeum vulgare* (NCBI). This mRNA is similar to a DNA binding protein in mouse and human [41]. This suggests it may act as a regulatory gene for stress response. EST to a WHE0854_F06_L12ZS is in the same bin and showed homology to a calcium/calmodulin-dependent protein kinase gene in Maize (NCBI). This gene has a role in stress signal transduction in plants. It also acts as a positive regulator for salt and ABA stress tolerance in plants [42]. Another EST in that bin, WHE2324_F12_L24ZS, was found to have similarity to a putative DNA topoisomerase I gene in rice. This gene plays a crucial role in stress adaptation of plants by altering gene expression [43]. This region would be of interest for further investigation.

Aquaporins are membrane-inserted water channel proteins that have been implicated in plant response to heat stress [44,45]. An analysis of wheat ESTs using the Thermorank program Li and Fang [46] showed that aquaporins are relatively heat labile (data not shown). Forrest and Bhave [47] physically mapped aquaporin loci in wheat and placed loci all group 2, group 4 and group 6 chromosomes as well as 3D, 5B, 7A and 7B. The aquaporins on 7A mapped to deletion bin 7AS 0–0.45 while the markers associated with the QTL in this study mapped to 7AL 0–0.31. Therefore, the aquaporins on 7A are not likely to be responsible for the trait. They did, however, map aquaporin genes to the same deletion bin as one of the flanking markers for the 6A QTL in this study (6AS 0–0.55). The QTL on 6A affected TMD and SCC but not PMD. The QTL on 2B was associated with PMD and does correspond to the physical region containing aquaporin genes, though the physical region encompasses most of the chromosome. An effort to understand what, if any, role aquaporins play in thermotolerance appears warranted, though it is unlikely to explain the majority of the effects observed in this study.

The epistatic region of chromosome 7A was found almost 20 cM away from the detected QTL of all three traits. This is most likely to be a distinct QTL rather than the existing one. The positive additive effect of that region (55 cM) was not significant, might be because of lack of marker information in that region of the linkage group (Figures 5 and 6). Concomitantly, epistatic region of chromosome 1B (25 cM) was found almost 13 cM away from the detected QTL of SCC (Figures 5 and 6). As SCC and PMD were found to be strongly correlated, the epistatic region of chromosome 1B might be different from the detected QTL in this study.

Transgressive segregation was observed in this population, meaning that both parents contribute alleles to the phenotype. Saadalla et al. [39] observed transgressive segregation for membrane thermostability. Even though there was transgressive segregation in the population, we failed to detect beneficial alleles from the sensitive parent. This could be due to to the smaller size of the population and relatively sparse marker coverage throughout the genome. Only 11.96% of our markers were mapped to the D genome. Most groups in the D genome were small. This might have prevented us from capturing alleles contributed from the sensitive parent.

The overall level of polymorphism in this population is surprisingly low. This may be attributed to the unknown pedigree of Ventnor, with Australian winter wheat background. Furthermore, it is possible that Ventnor contains US Great Plains wheat derived through international germplasm exchange; if that is the case, the genetic diversity between the two parents would be low.

In our study, five QTL regions that significantly influenced TMD, SCC and PMD were detected on chromosomes 6A, 7A, 1B, 2B and 1D. The SSR markers *Xbarc121* and *Xbarc49* for all three traits on chromosome 7A, and *gwm18 and Xbarc113* for SCC on chromosome 1B and 6A, respectively were found close to the QTL. Along with the SSRs, five GBS Bin markers *Bin747, Bin 1596, Bin 178, Bin 81* and *Bin 1130* were also found strongly associated with TMD, SCC and PMD. BIN markers have been called from SNPs (Additional file 2), and the sequence information of these relevant SNPs can be used for further exploration of marker assisted selection. The AFLP marker, *XCGT.AGCT347* would be a good target for conversion to a more user friendly marker.

## Conclusions

Heat tolerance is a complex trait and influenced by different component traits. Our study suggests that membrane damage and chlorophyll content are very closely correlated when plants are exposed to a long period of heat stress, and are most likely under similar genetic control. The neighboring markers in the identified QTL may play an important role in marker assisted breeding for heat tolerance in wheat.

### Future research goals

The authors realize that it is important to observe the performance of this population under field conditions to validate the findings of this study. However, due to the nature of the population (spring/winter wheat cross), it would be difficult to conduct a validation study under field conditions due to the lack of winter hardiness of RILs, which would result in cold damage to many RILs and would create undesirable variations in the study. Considering this point, authors have initiated a process of transferring these QTL into different winter wheat backgrounds by using the back cross breeding method. Once the backcross populations are developed, their performance will be tested under field conditions and the results will be published in a peer reviewed journal.

## Additional files

Additional file 1:
**SNP sequence and Bin information.**
Additional file 2:
**All linkage maps.**
