# Supplementary figures and images for: Mapping QTL for the traits associated with heat tolerance in wheat (Triticum aestivum L.)
Source: BMC Genet. 2014 Nov 11;15:97. doi: 10.1186/s12863-014-0097-4 (PMC4234900; doi:10.1186/s12863-014-0097-4)

**
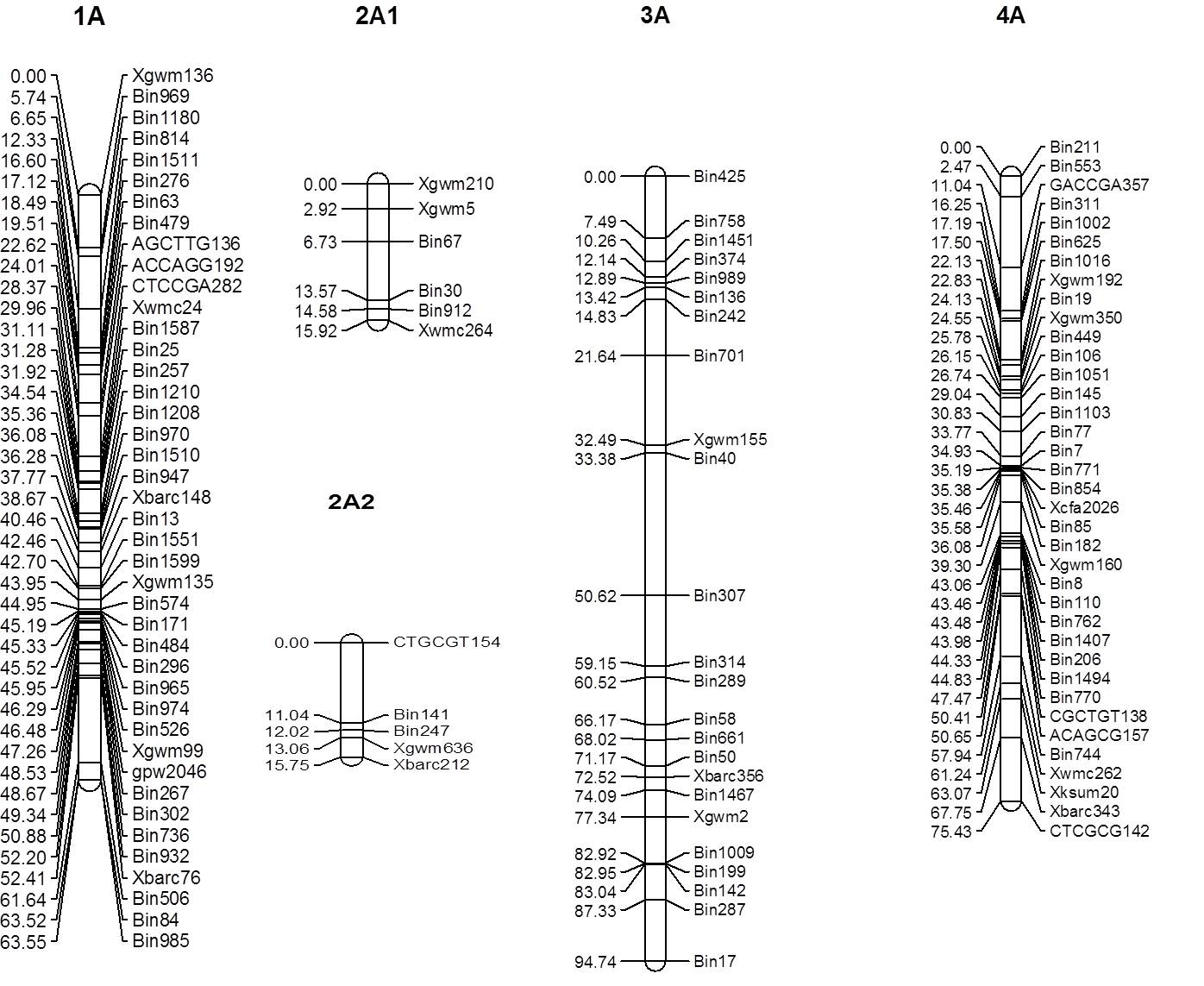
All 22 linkage groups along with QTL are presented in this file**


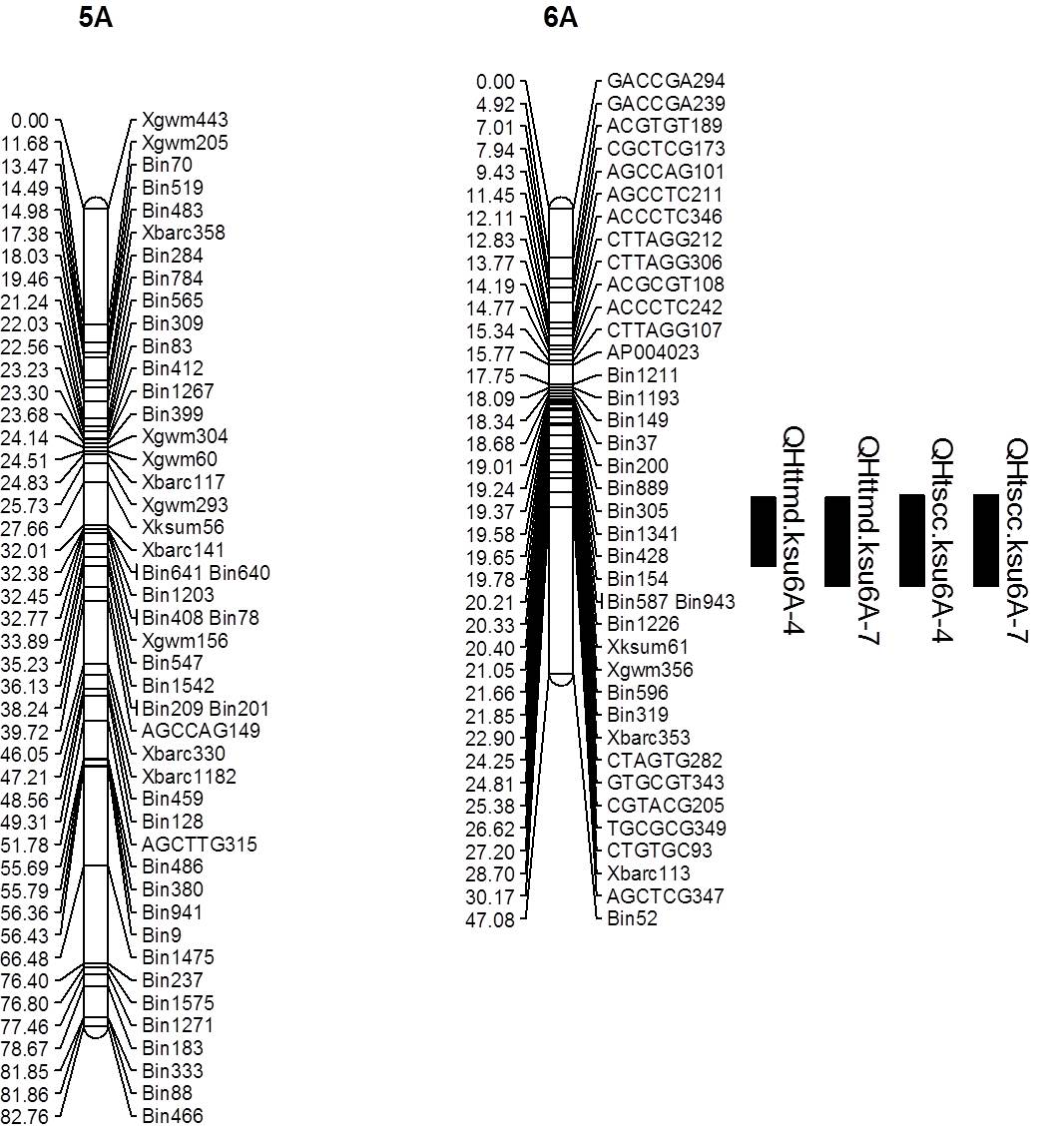


**
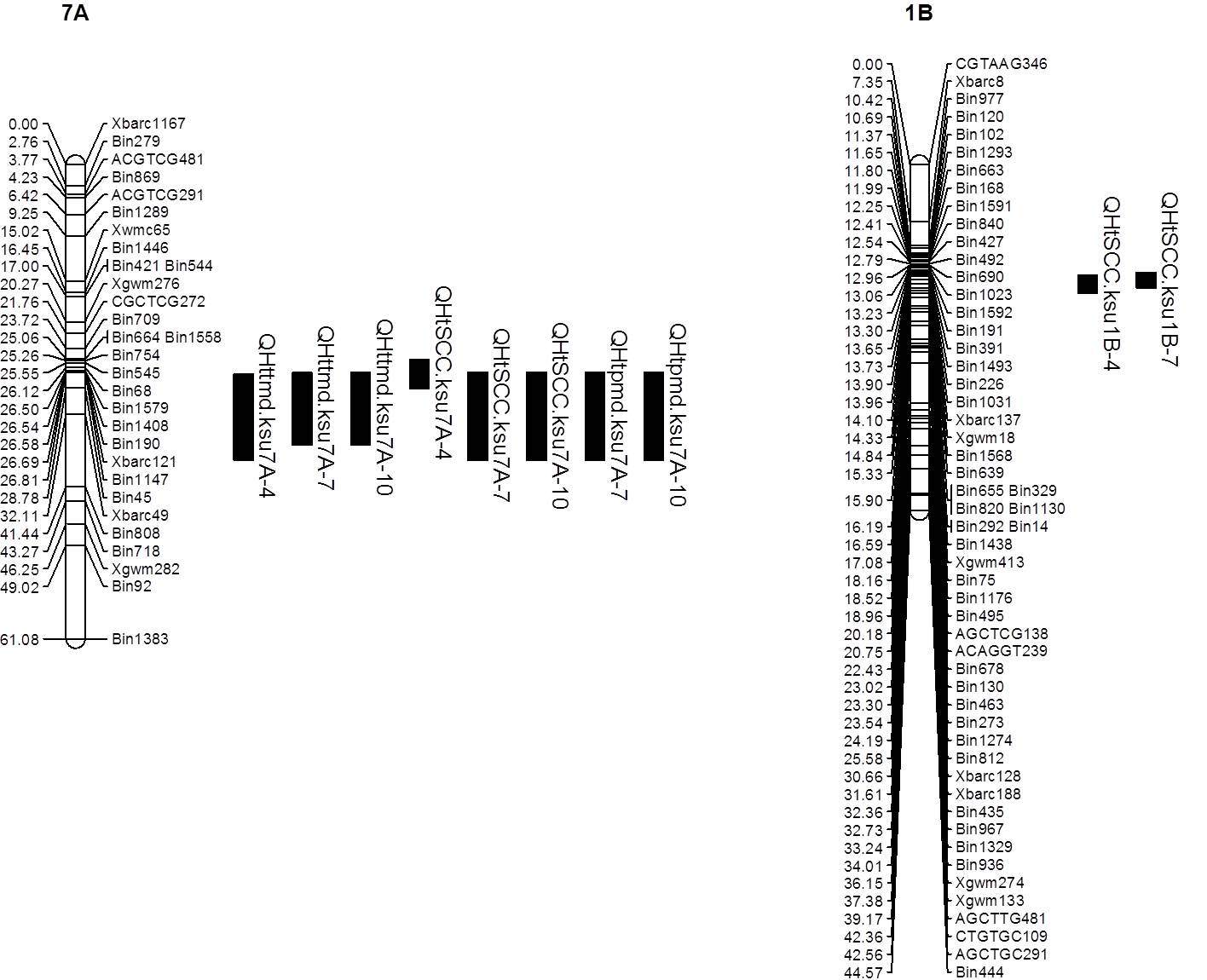
**

**
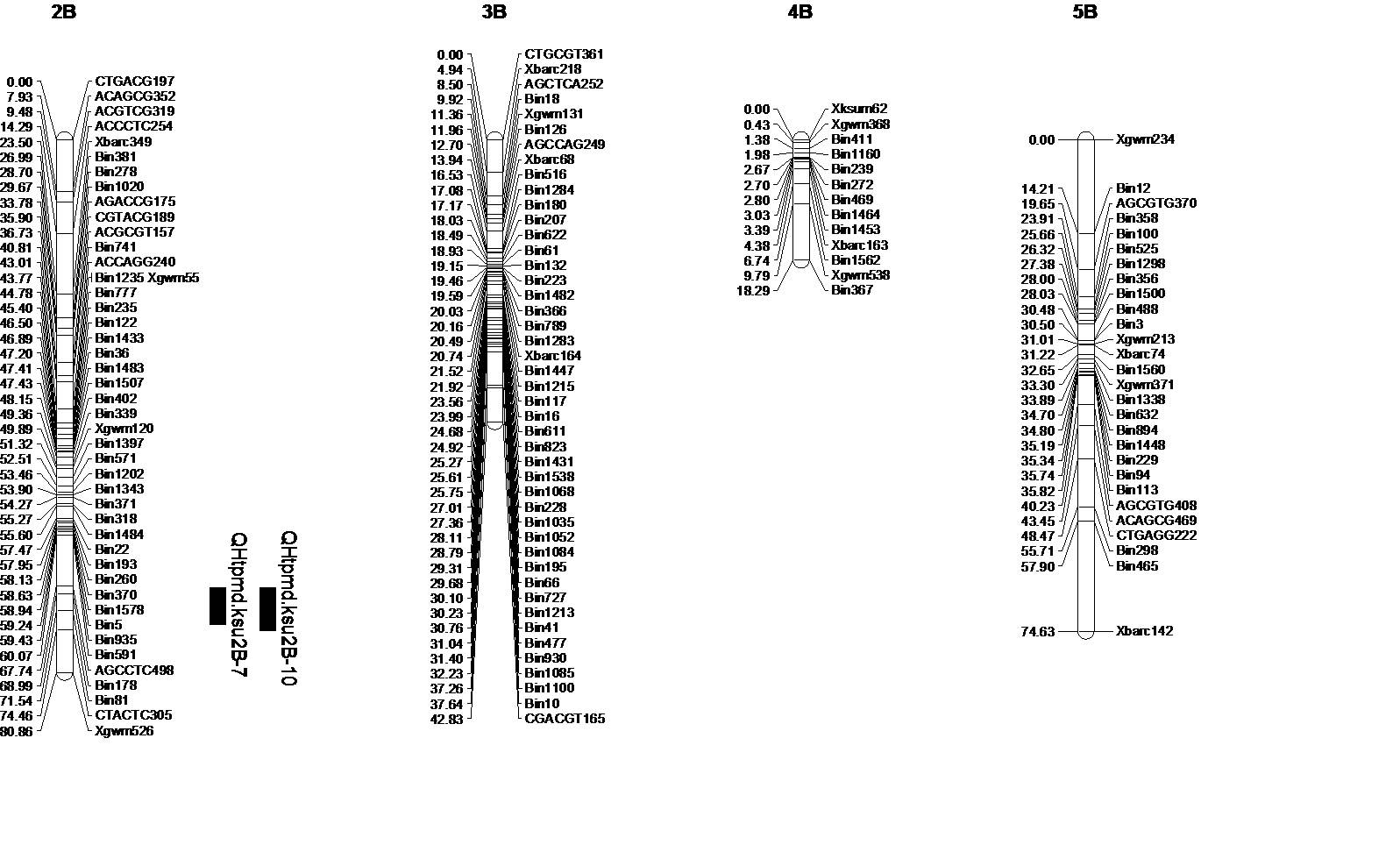
**


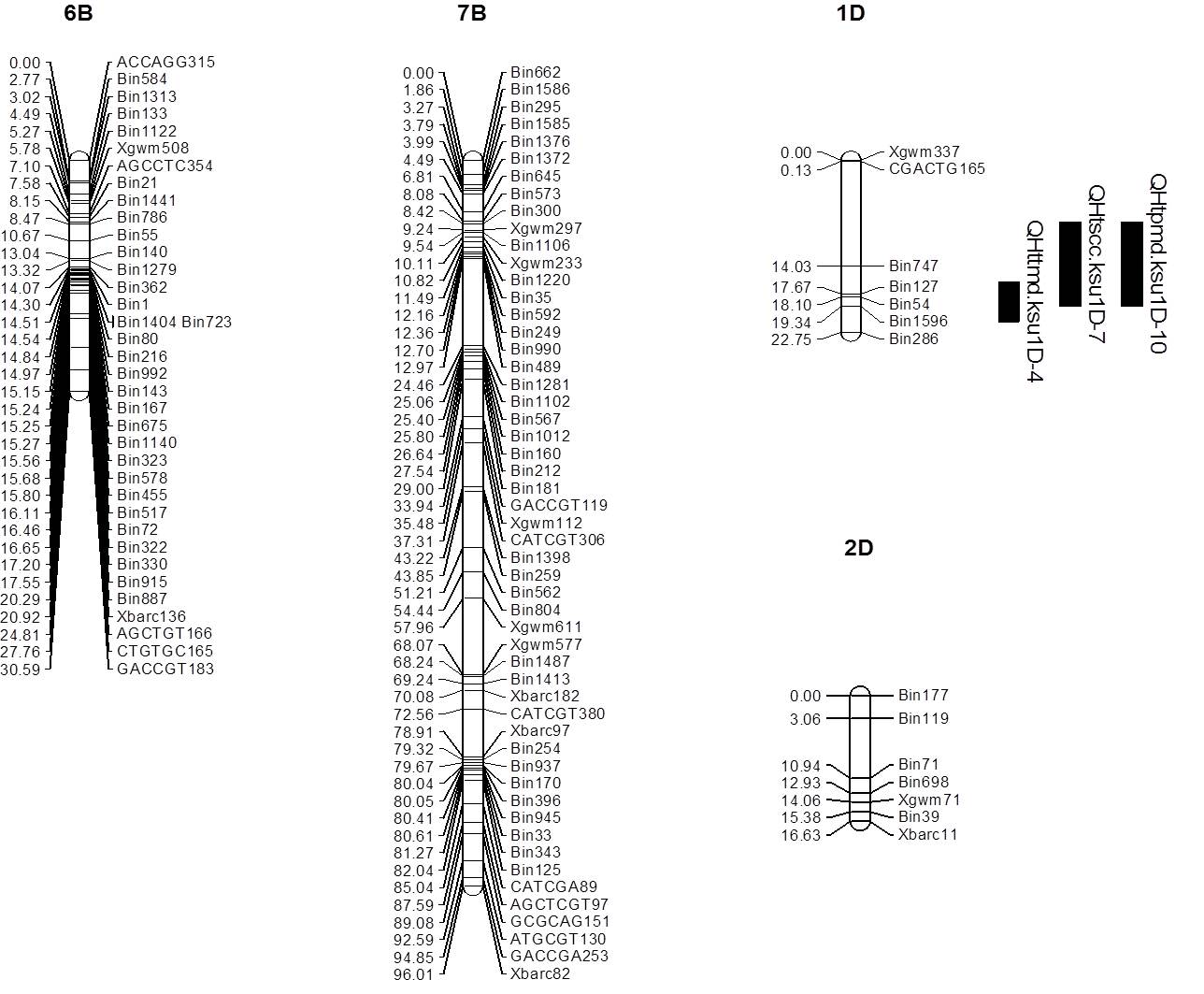


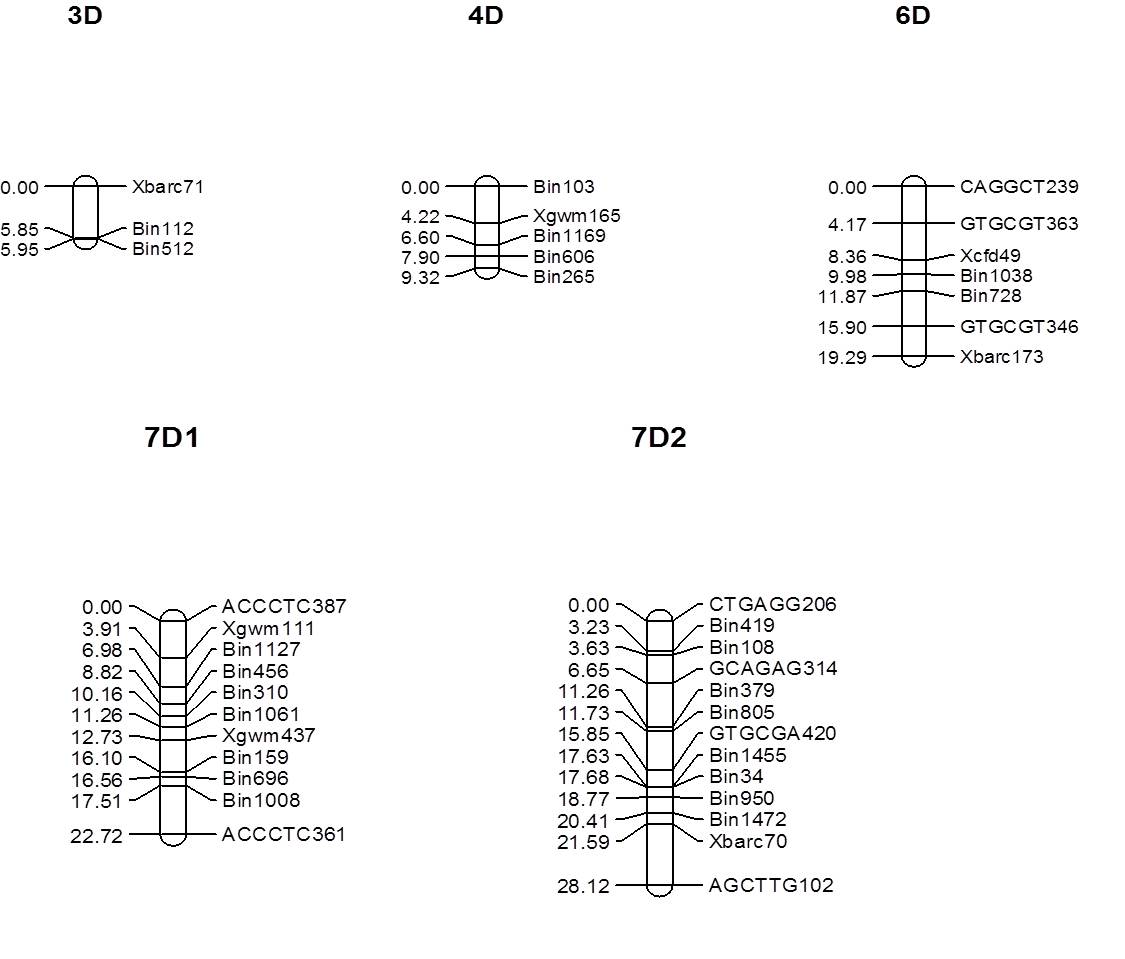

Supplement: Additional file 2: — All linkage maps. [file 12863_2014_97_MOESM2_ESM.docx]
